# Role of membrane Hsp70 in radiation sensitivity of tumor cells

**DOI:** 10.1186/s13014-015-0461-1

**Published:** 2015-07-22

**Authors:** Naoya Murakami, Annett Kühnel, Thomas E. Schmid, Katarina Ilicic, Stefan Stangl, Isabella S. Braun, Mathias Gehrmann, Michael Molls, Jun Itami, Gabriele Multhoff

**Affiliations:** Department of Radiation Oncology, Klinikum rechts der Isar, Technische Universität München, Munich, Germany; Department of Radiation Oncology, National Cancer Center Hospital, Tokyo, Japan; Clinical Cooperation Group - Innate Immunity in Tumor Biology, Institute of Biomedical Imaging (IBMI), Helmholtz Zentrum München, Munich, Germany

**Keywords:** Heat shock protein 70, Membrane localization, Apoptosis, X-ray irradiation, Radiation resistance

## Abstract

**Background:**

The major stress-inducible heat shock protein 70 (Hsp70) is frequently overexpressed in the cytosol and integrated in the plasma membrane of tumor cells via lipid anchorage. Following stress such as non-lethal irradiation Hsp70 synthesis is up-regulated. Intracellular located Hsp70 is known to exert cytoprotective properties, however, less is known about membrane (m)Hsp70. Herein, we investigate the role of mHsp70 in the sensitivity towards irradiation in tumor sublines that differ in their cytosolic and/or mHsp70 levels.

**Methods:**

The isogenic human colon carcinoma sublines CX^+^ with stable high and CX^−^ with stable low expression of mHsp70 were generated by fluorescence activated cell sorting, the mouse mammary carcinoma sublines 4 T1 (4 T1 ctrl) and Hsp70 knock-down (4 T1 Hsp70 KD) were produced using the CRISPR/Cas9 system, and the Hsp70 down-regulation in human lung carcinoma sublines H1339 ctrl/H1339 HSF-1 KD and EPLC-272H ctrl/EPLC-272H HSF-1 KD was achieved by small interfering (si)RNA against Heat shock factor 1 (HSF-1). Cytosolic and mHsp70 was quantified by Western blot analysis/ELISA and flow cytometry; double strand breaks (DSBs) and apoptosis were measured by flow cytometry using antibodies against γH2AX and real-time PCR (RT-PCR) using primers and antibodies directed against apoptosis related genes; and radiation sensitivity was determined using clonogenic cell surviving assays.

**Results:**

CX^+^/CX^−^ tumor cells exhibited similar cytosolic but differed significantly in their mHsp70 levels, 4 T1 ctrl/4 T1 Hsp70 KD cells showed significant differences in their cytosolic and mHsp70 levels and H1339 ctrl/H1339 HSF-1 KD and EPLC-272H ctrl/EPLC-272H HSF-1 KD lung carcinoma cell sublines had similar mHsp70 but significantly different cytosolic Hsp70 levels. γH2AX was significantly up-regulated in irradiated CX^−^ and 4 T1 Hsp70 KD with low basal mHsp70 levels, but not in their mHsp70 high expressing counterparts, irrespectively of their cytosolic Hsp70 content. After irradiation γH2AX, Caspase 3/7 and Annexin V were up-regulated in the lung carcinoma sublines, but no significant differences were observed in H1339 ctrl/H1339 HSF-1 KD, and EPLC-272H ctrl/EPLC-272H HSF-1 KD that exhibit identical mHsp70 but different cytosolic Hsp70 levels. Clonogenic cell survival was significantly lower in CX^−^ and 4 T1 Hsp70 KD cells with low mHsp70 expression, than in CX+ and 4 T1 ctrl cells, whereas no difference in clonogenic cell survival was observed in H1339 ctrl/H1339 HSF-1 KD and EPLC-272H ctrl/ EPLC-272H HSF-1 KD sublines with identical mHsp70 but different cytosolic Hsp70 levels.

**Conclusion:**

In summary, our results indicate that mHsp70 has an impact on radiation resistance.

## Background

Apart from surgery and chemotherapy, radiotherapy is one of the three key treatment modalities to treat localized solid tumors. The major goal of therapeutic irradiation is to inflict deleterious damage selectively in tumor cells. While some cancer cells are directly killed by irradiation, others appear to activate mechanisms of resistance. Similar to other stress stimuli such as hyperthermia, hypoxia, reactive oxygen species (ROS), heavy metals, cytotoxic drugs, glucose deprivation also ionizing irradiation induces a complex stress reaction in the exposed tumor and normal tissues [1, 2]. Heat shock protein 70 (Hsp70), one of the major stress-inducible members of the 70 kDa stress protein family (HSP70) whose expression is mainly regulated by Heat Shock Factor 1 (HSF-1) consists of at least 8 homologous members, exerts tumorigenic functions by sustaining proliferative cell signaling, increasing invasive and metastatic activity and migration and by preventing apoptotic signaling [3, 4]. Hsp70 is frequently constitutively overexpressed in the cytosol and present on the plasma membrane of many different tumor types [5–7] to promote cancer cell survival, tumorigenicity and anti-apoptotic activities, such as interfering with the apoptosis signal regulating kinase 1 (ASK1) and the co-chaperone CHIP [5–11], blocking BAX translocation to the mitochondria [12] or by interfering with lysosomal membranes and thereby inhibiting their permeabilization [13]. Apart from its intracellulat localization, Hsp70 can be transported to and anchored on the plasma membrane of tumor, but not normal cells, via tumor-specific lipid vesicular transport which is not completely unravelled [14]. Membrane Hsp70-positive tumors have been shown to actively release Hsp70 in exosomes [14, 15] that can fuse with the plasma membrane. Since normal cells do not present Hsp70 on their cell surfaces, mHsp70 serves as a tumor-specific targeting structure for *in vivo* imaging [16, 17], and lipid-bound Hsp70 in the blood might provide a novel tumor biomarker in liquid biopsies [14, 15].

As mentioned before, cytosolic Hsp70 exerts cytoprotective properties by interfering with anti-apoptotic signaling pathways [18]. In mammalian cells, apoptosis can be caused by either intrinsic or extrinsic pathways [19] whereby apoptotic factors such as cytochrome *c* which are released by mitochondria with a disturbed membrane potential induce the intrinsic pathway [20, 21], and the binding of extracellular protein death ligands of the tumor necrosis factor (TNF) family to pro-apoptotic death receptors (DRs) on the cell surface can initiate the extrinsic apoptotic cascade [20].

Overexpression of Hsp70 can provide tumor cells with a selective survival advantage in part due to its ability to inhibit multiple pathways of cell death, including both intrinsic and extrinsic apoptosis [10, 22, 23]. Hsp70 can bind directly to the pro-apoptotic Bcl-2 family member BAX, which is part of the intrinsic apoptosis pathway and thus prevents its activation and translocation to the mitochondria [24, 25]. Hsp70 can also interact with death receptors DR4 and DR5 of the extrinsic apoptotic pathway and thus inhibits the assembly of the death-inducing signaling complexes [26]. Therefore, inhibition of cytosolic Hsp70 provides a promising concept in anti-cancer therapies. It also has been described that mHsp70-positive tumor cells are better protected against ionizing irradiation compared to their mHsp70-negative counterparts [27]. Herein, we want to study the impact of cytosolic versus mHsp70 in the radiosensitivity of four isogenic tumor cell systems.

## Materials and methods

### Cells and cell culture

Three human and one mouse carcinoma subline of different origin were used in the study. The size of mouse carcinoma cells significantly smaller than that of the human tumor cell lines. The human adeno colon carcinoma cell line CX-2 (Tumorzellbank, DKFZ Heidelberg, Germany) gave rise to the sublines CX^+^ with a stable high and CX^−^ with a low mHsp70 expression after fluorescence activated cell sorting [27, 28]. The HSF-1 knock-down (HSF-1 KD) and ctrl human lung cancer cell lines H1339 (small cell lung carcinoma, SCLC) and EPLC-272H (non-small cell lung carcinoma, NSCLC; kindly provided by Prof. Rudolf Huber, Dpt. of Pneumonology, University Munich, Germany) as well as the CX^+^/CX^−^ sublines were cultured in Roswell Park Memorial Institute (RPMI)1640 medium (GIBCO, Eggenstein, Germany) supplemented with 10 % *v/v* heat-inactivated fetal calf serum (FCS) (PAA, Pasching, Austria), 1 % *v/v* antibiotics (100 IU/ml penicillin, 100 μg/ml streptomycin, GIBCO), 2 mM L-glutamine (GIBCO) and 1 mM sodium pyruvate (GIBCO). All adherent growing tumor cells were trypsinized for less than 3 min with trypsin-ethylene diamine-tetra-acetic acid (EDTA) (GIBCO), and single cell suspensions were seeded at constant cell densities of 1.5 × 10^6^ cells in 15 ml fresh medium in T-75 ventilated culture flasks (Greiner, Nuertingen, Germany).

For knock-down of Hsp70 in the lung carcinoma cells H1339 and EPLC-272H HSF1 RNAi-Ready pSIREN-RetroQ vectors with a puromycin resistance (BD Biosciences) was used. Target sequence for HSF-1 small interfering RNA was 5′-TATGGACTCCAACCTGGATAA-3′ [29]. Retroviruses were produced by transfection of Phoenix cells with pSIREN-RetroQ/HSF1 shRNA (shHSF1) or pSIREN-RetroQ (control) (kindly provided by Profs. J. Yaglom and M. Sherman, Boston University School of Medicine, USA) using Ca-phosphate. Tumor cells were infected with virus containing supernatants in the presence of 10 μg/ml polybrene. Selection was performed with 2 μg/ml puromycin.

The mouse mammary carcinoma derived 4 T1 cell line (ATCC®CRL-2539™, American Type Culture Collection, Manassas, USA) was used to knock-down Hsp70 by the CRISPR/Cas9 system [17, 30]. The RNA-guided Cas9 nuclease [31], derived from *Streptococcus pyogenes* was used to induce specific DSBs in regions of the chromosome that are coding for Hsp70 (HSPA1A/B). After DSBs were introduced to specific loci, damaged DNA was repaired by non-homologous end joining (NHEJ) [32] which resulted in shift of the sequence and a knock-down (KD) of the targeted gene was achieved [33]. By using this procedure the subline 4 T1 Hsp70 KD was generated which showed a significant but not complete reduction in cytosolic and mHsp70. As a control, 4 T1 cells were transfected with a control vector 4 T1 (ctrl). To obtain a complete Hsp70 knock-out a second and third treatment cycle using the CRISPR/Cas9 system was necessary [17]. The 4 T1 sublines were cultured in Roswell Park Memorial Institute 1640 medium (GIBCO, Eggenstein, Germany) supplemented with 10 % *v/v* heat-inactivated fetal calf serum (FCS) (GIBCO), 1 % *v/v* antibiotics (penicillin-streptomycin, GIBCO), 2 mM L-glutamine (GIBCO), non-essential amino acids, and β-mercaptoethanol. All adherent tumor cells were trypsinized for 5 min with trypsin-ethylene diamine-tetra acetic acid (EDTA) (GIBCO), and single cell suspensions were seeded at constant cell densities of 1.0 × 10^6^ cells in 20 ml fresh medium in T-75 ventilated culture flasks (Greiner, Nuertingen, Germany). Following exposure to stress the 4 T1 Hsp70 KD cell line showed a moderate up-regulation of Hsp70 in the cytosol which was less pronounced than that of 4 T1 ctrl cells (data not shown).

All tumor cell sublines were routinely checked and determined as negative for mycoplasma contamination.

### SDS-PAGE, western blot analysis, ELISA

Cytosolic proteins were obtained as described previously [1]. Briefly, 2 × 10^6^ cells were lysed in 10 mM Tris-buffered saline (TBST buffer, pH 7.5) containing 1 % Nonidet P-40 (NP-40; Sigma Aldrich, St. Louis, MO, USA) on ice for 45 min. Non-soluble material was pelleted by centrifugation at 10,000 g and discarded. Protein concentration was determined with the bicinchoninic acid method (BCA Protein Assay Kit; Pierce, Thermo Fisher Scientific Inc., Rockford, IL, USA). Equal protein amounts (20 μg) were subjected to the 10 % sodium dodecyl sulfate-polyacrylamide gel (SDS-PAGE) following a standard protocol [34]. 10 ng and 25 ng of recombinant Hsp70 protein [35] were loaded as positive control and for protein quantification. After SDS-PAGE, the proteins were transferred to nitrocellulose membranes (Millipore Corp., Bedford, MA, USA) and/or PVDF membranes following a standard protocol [36]. Non-specific binding of the membranes was blocked with 5 % skim milk in PBS supplemented with 1 % Tween-20. Blots were incubated with the Hsp70 primary antibody (cmHsp70.1, IgG1; multimmune, Munich, Germany) and a β-actin antibody (A5316, Sigma-Aldrich) at 4 °C for 14 h. After two washing steps, membranes were incubated with a secondary antibody (goat anti-mouse IgG peroxidase-conjugated; Promega, Madison, WI, USA) at room temperature for 1 h. All antibodies were diluted in 1 % skim milk in PBS supplemented with 1 % Tween-20. Immune complexes were detected using the ECL detection system (Amersham Biosciences, Buckinghamshire, UK). Blots were imaged digitally (ChemiDoc Touch Imaging System, Biorad Laboratories, Hercules, CA, USA) and quantified using Image Lab Software (Biorad Laboratories) with automatic settings. The absolute amount of Hsp70 was determined by the ratio of the ß-actin-normalized integrated volumes of bands, related to Hsp70 positive controls with known amounts which were used as internal standards.

Hsp70 concentrations were verified by ELISA (R&D systems) in cell lysates following the manufacturer’s recommendations. Hsp70 concentrations were calculated relative to the total protein content of each sample.

### Irradiation of tumor cells

CX sublines were irradiated with a single dose of 0 Gy (sham) to 20 Gy, using the Gulmay RS225A irradiation machine (Gulmay Medical Ltd., Camberley, UK) at a dose rate of 0.90 Gy/min (15 mA, 200 keV). A dose of 20 Gy was chosen for γH2AX and Caspase 3/7 and Annexin V assays because concentrations below did not result in statistically significant results but showed the same trend. 4 T1, H1339 and EPLC-272H cell sublines were irradiated with a single dose of 0 Gy (sham) to 6 Gy with same irradiation equipment as used for the CX sublines.

### Flow cytometry of mHsp70

Single cell suspensions of untreated and treated CX, 4 T1, H1339 and EPLC-272H sublines were collected at different time-points after irradiation. A sample of 0.4 × 10^6^ cells was washed once with 10 % FCS in phosphate-buffered saline (PBS) and incubated with fluorescein isothiocyanate (FITC)-conjugated mouse monoclonal antibody specific for mHsp70 (cmHsp70.1, IgG1; multimmune GmbH, Munich, Germany) or a FITC-labeled isotype-matched IgG-negative control antibody (code 345815; BD Biosciences, NJ, USA) on ice in the dark for 30 min. Only propidium iodide-negative, viable cells were gated and the mean fluorescence intensity (mfi) of antibody-bound cells was analyzed by a FACSCalibur flow cytometer (Becton Dikinson, Heidelberg, Germany). The mfi is a relative value of the total intensity of the signal derived from cmHsp70.1-FITC antibody stained, viable cells (50,000) subtracted by the intensity of the signal intensity which is derived by viable cells stained with an isotype-matched IgG1-FITC control antibody. Fluorescence data were plotted by using CellQuest software (Becton Dickinson, Heidelberg, Germany).

### Flow cytometry of үH2AX

DSBs were assessed by using γH2AX (phosphorylated Histone H2AX) antibody. The single cell suspensions of either sham irradiated and/or irradiated CX, 4 T1, H1339 and EPLC-272H sublines were collected 1 h after irradiation using trypsin and by mechanical disruption. Single cell suspensions containing 0.6 × 10^6^ cells were washed in 0.5 ml PBS and fixed with 70 % ethanol/acetic acid (3:1). Fixed cells were kept at −20 °C for up to two weeks before analysis. After removal of the fixative, cell pellets were re-suspended with 1 ml of 0.15 % Triton X-100 solution and incubated on ice with anti-phospho-Histone H2AX, Alexa Fluor 488 conjugate (Novus Biologicals). The mfi of cells that did bind the antibody was analyzed by flow cytometry.

### Quantitative analysis of gene expression

48 h after irradiation total RNA was extracted from cell pellet containing 1.0 × 10^6^ cells of CX or 4 T1 cells with MasterPureTM RNA Purification Kit (Epicentre, Madison, USA). The concentration of RNA was measured by a Nanodrop™ spectrophotometer at 260 nm. RNA was reversely transcribed into cDNA using High Capacity cDNA Reverse Transcription Kits (Life Technologies GmbH, Darmstadt, Germany). The resulting cDNA was subjected to quantitative RT-PCR using primers directed towards Caspase 3, Caspase 7, Caspase 8, Caspase 9, BAD and BAX. Actin B (ActB) was used as a house keeping gene. The reaction mix was prepared according to the standard protocol of the kit. RT-PCR was carried out with a LightCycler® 480 (Roche Diagnostics, Mannheim, Germany) with a standard thermal profile. Transcriptional changes were calculated with delta Ct method and normalized with the house keeping gene. Mean values were obtained from triplicate samples.

### Expression of active Caspase 7, phosphorylated (p)BAD and BAX

Cell lysates (50 μg/ml) were subjected to SDS-PAGE (12.5 %) and after transfer, membranes [36] were incubated with a primary rabbit-anti-active Caspase 7, rabbit-anti-phospho-BAD (1:1000; Ser^136^), and rabbit-anti-BAX (1:1000; Cell Signaling Technology, Beverly, MA, USA) antibody. Following washing, the nitrocellulose (NC) membranes were incubated with a horseradish peroxidase (HRP) conjugated secondary anti-rabbit IgG and detected using the ECL system, as described above.

### Flow cytometry of active Caspase 3/7 and Annexin V

Apoptosis was assessed in all tumor sublines using CellEvent® Caspase 3/7 Green Detection Reagent (Life Technologies, Darmstadt, Germany). Single cell suspensions of sham irradiated and irradiated tumor cells were collected 24 and 48 h after irradiation using trypsin and mechanical disruption. The single cell suspension containing 0.4 × 10^6^ were washed in 1 ml PBS and incubated with CellEvent^®^ Caspase 3/7 Green Detection Reagent and SYTOX AADvanced® dead cell stain solution. The percentage of antibody-bound cells among viable cells was determined on a flow cytometer.

Apoptosis was determined in CX^+^/CX^−^ and 4 T1 ctrl/4 T1 Hsp70 KD tumor sublines also by Annexin V-FITC staining. After washing with Annexin V binding buffer cells were incubated with Annexin V-FITC (Roche Diagnostics, Mannheim, Germany) for 15 min at room temperature. After another washing step in PBS/10 % *v/v* FCS and 2.5 mM CaCl_2_, propidium iodide (PI) was added for 1 min. Then cells were analyzed on a FACSCalibur flow cytometer (BD).

### Clonogenic cell survival assay

Single cell suspensions were seeded onto 12-well plates. CX tumor sublines were irradiated with 0, 2, 4, 6, and 10 Gy and 4 T1, H1339, and EPLC-272H tumor sublines were irradiated with 0, 2, 4, and 6 Gy at the Gulmay RS225A irradiation machine (Gulamy Medical Ltd., Cambereley, UK). A dose of 20 Gy resulted in too few colonies and thus could not be analyzed (data not shown). Cells were fixed in ice cold methanol after reaching colonies containing more than 50 cells and stained with 0.1 % crystal violet. All colonies with more than 50 cells were counted automatically using a Bioreader® (Bio-Sys GmbH, Karben, Germany). To create a radiation survival curve, the survival fraction at each radiation dose was normalized to that control which was sham-irradiated with 0 Gy.

### Statistical analysis

Mean value was calculated and presented with standard deviation. The Student *t-*test was used to evaluate the relevance between variables. A *p*-value of ≤0.05 was considered as statistically significant.

## Results

### Comparison of cytosolic and mHsp70 levels in isogenic tumor sublines

**Fig. 1:**
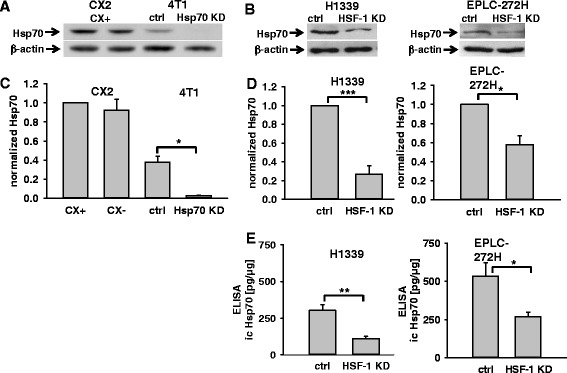
Representative Western blot analysis of cytosolic Hsp70 in CX^+^/CX^−^ (**a**), 4 T1 ctrl/4 T1 Hsp70 KD (**a**), H1339 ctrl/H1339 HSF-1 KD, EPLC-272 ctrl/EPLC-272H HSF-1 KD (**b**) tumor sublines under non-stressed conditions. 20 μg of cytosolic proteins were administered to each lane of a SDS/PAGE and blotted onto nitrocellulose membranes. Hsp70 and ß-actin were detected using primary antibodies, an appropriate HRP-conjugated secondary antibody, followed by visualization with ECL kit. β-actin and recombinant Hsp70 protein (10 ng, 25 ng; data not shown) were used as loading control and for normalization. Normalized Hsp70 values of CX^+^/CX^−^ and 4 T1 ctrl/4 T1 Hsp70 KD (**c**), H1339 ctrl/H1339 HSF-1 KD and EPLC-272H ctrl/EPLC-272H HSF-1 KD (**d**, **e**) cell lysates, as determined by Western blot analysis (**c**, **d**) and ELISA of cell lysates (**e**). Bars represent the mean values of *n* = 3-4 independent experiments. Significance: *p ≤ 0.05, **p ≤ 0.01, ***p ≤ 0.001

**Fig. 2:**
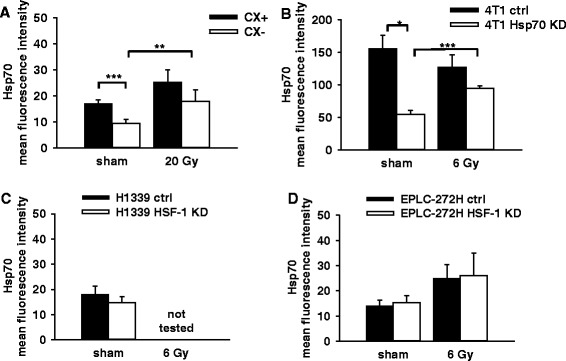
Mean fluorescence intensity (mfi) of mHsp70 in CX^+^/CX^−^ cells 48 h after sham (0 Gy) and 20 Gy irradiation (**a**, *n* = 3-4), 4 T1 ctrl/4 T1 Hsp70 KD cells after sham (0 Gy) and 6 Gy irradiation (**b**, *n* = 3-4), H1339 ctrl/H1339 HSF-1 (**c**, *n* = 4) after sham (0 Gy) and EPLC-272H ctrl/EPLC-272H HSF-1 KD (**d**, *n* = 6) after sham (0 Gy) and 6 Gy irradiation. Bars represent the mean values and the corresponding standard error of the mean (SEM) of *n* = 3-6 independent experiments. Significance: *p ≤ 0.05, **p ≤ 0.01, ***p ≤ 0.001

We investigated cytosolic and mHsp70 levels in isogenic human colon carcinoma sublines CX^+^/CX^−^, mouse mammary carcinoma cell lines 4 T1 ctrl/4 T1 Hsp70 knock-down (4 T1 Hsp70 KD), human lung carcinoma cell sublines H1339 ctrl/H1339 HSF-1 KD and EPLC-272H ctrl/EPLC-272H HSF-1 KD. Figure 1 shows representative Western blots of tumor cell lysates of all cell lines under non-stressed conditions using cmHsp70.1 antibody for the detection of Hsp70. A defined amount of recombinant Hsp70 protein (10 ng and 25 ng) was loaded onto the gel for quantification and β-actin was used as a loading control and for normalization. Although CX^+^ (36.4 ± 4.8 ng) and CX^−^ (32.9 ± 5.0 ng) tumor sublines revealed similar cytosolic Hsp70 levels (Fig. 1a and c), the tumor sublines differed significantly in their mHsp70 expression under non-stressed conditions (sham; Fig. 2a). Following irradiation at 20 Gy the mHsp70 expression was significantly up-regulated on CX^−^ but not CX^+^ tumor cells (20 Gy; Fig. 2a). A lower irradiation dose of 10 Gy showed a similar trend with respect to the mHsp70 expression on CX^−^ cells but did not reach statistical significance (data not shown).

No differences in the cytosolic Hsp70 levels were detected in 4 T1 cells that had been transfected with a control vector (ctrl) versus 4 T1 wild type (WT) cells (data not shown). A CRISPR/Cas9 Hsp70 knock-down (4 T1 Hsp70 KD) revealed significantly lower cytosolic Hsp70 levels than in 4 T1 ctrl (Fig. 1a and b) and 4 T1 WT cells (data not shown), and mHsp70 levels were significantly higher in 4 T1 ctrl versus 4 T1 Hsp70 KD cells under non-stressed conditions (sham; Fig. 2b). Following irradiation at 6 Gy the mHsp70 expression was significantly up-regulated in 4 T1 Hsp70 KD cells but not in 4 T1 ctrl cells (6 Gy; Fig. 2b). A lower irradiation dose of 4 Gy showed a similar trend but the values did not reach statistical significance (data not shown). 4 T1 mouse mammary carcinoma cells showed lower Hsp70 levels in the cytosol than human CX^+^/CX^−^ cells (Fig. 1a). Although the density of Hsp70 molecules on the cell surface is similar in mouse and human tumor cells, the mfi appears to be higher in 4 T1 cells due to their smaller size.

A siRNA knock-down of HSF-1 in H1339 and EPLC-272H lung carcinoma cells resulted in a significant down-regulation of Hsp70 in the cytosol of these tumor sublines, as determined by Western blot analysis (Fig. 1d) and ELISA (Fig. 1e). However, the reduced cytosolic Hsp70 levels induced by HSF-1 knock-down did not affect the mHsp70 levels, under non-stressed conditions (Fig. 2c and d) and following irradiation at 6 Gy (Fig. 2d).

### Comparison of radiation-induced DNA double strand breaks (DSBs) in tumor sublines with different cytosolic and mHsp70 levels

**Fig. 3:**
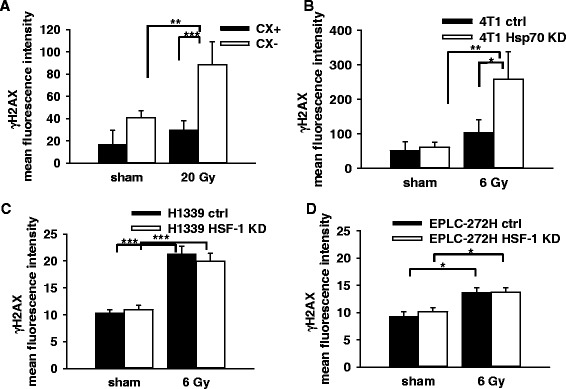
Mean fluorescence intensity (mfi) of үH2AX foci in CX^+^/CX^−^ cells 1 h after sham (0 Gy) and 20 Gy irradiation (**a**), and 4 T1 ctrl/4 T1 Hsp70 KD cells (**b**), H1339 ctrl/H1339 HSF-1 cells (**c**), EPLC-272H ctrl/EPLC-272H HSF-1 KD cells (**d**) 1 h after sham (0 Gy) and 6 Gy irradiation, respectively (**b**, **c**, **d**). Bars represent the mean values and the corresponding standard error of the mean (SEM) of *n* = 3 independent experiments. Significance: *p ≤ 0.05, **p ≤ 0.01, ***p ≤ 0.001

A quantification of the γH2AX foci as a measure for DSBs was determined by flow cytometry one hour after irradiation of CX^+^/CX^−^ cells (Fig. 3a) with 0 Gy (sham) and 20 Gy, 4 T1 ctrl/4 T1 Hsp70 KD cells (Fig. 3b) with 0 Gy (sham) and 6 Gy, H1339 ctrl/H1339 HSF-1 KD (Fig. 3c) and EPLC-272H ctrl/EPLC-272H HSF-1 KD cells (Fig. 3d) with 0 Gy (sham) and 6 Gy. After irradiation a significant increase in γH2AX was observed in CX^−^ (40.58 ± 6.45 vs. 88.52 ± 14.19, *p* ≤ 0.01) and 4 T1 Hsp70 KD cells (60.64 ± 15.20 vs. 257.81 ± 32.04, *p* ≤ 0.01), whilst no statistically significant increase was observed in non-irradiated versus irradiated CX^+^ and 4 T1 ctrl (or WT cells, data not shown) with initially high mHsp70 expression. The γH2AX mfi values of irradiated CX^+^/CX^−^ (20 Gy; *p* ≤ 0.001) and 4 T1 ctrl/4 T1 Hsp70 KD (6 Gy; *p* ≤ 0.05) sublines also differed significantly. γH2AX did not differ significantly in the sublines H1339 ctrl/H1339 HSF-1 KD and EPLC-272H/EPLC-272H HSF-1 KD that show an identical mHsp70 expression (Fig. 3c and d). However, significant differences in үH2AX foci were observed in sham (0 Gy) and irradiated (6 Gy) lung cancer sublines were compared (Fig. 3c and d).

### Comparison of radiation-induced apoptosis in tumor sublines with different cytosolic and mHsp70 levels

**Fig. 4:**
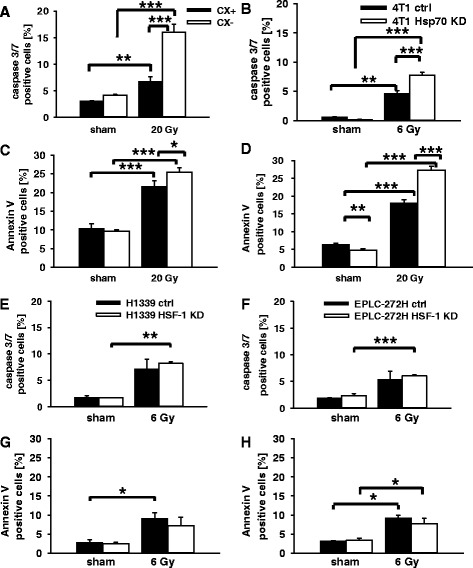
Percentage of apoptotic cells as assessed by active Caspase 3/7 (**a**, **b**, **e**, **f**) and Annexin V (**c**, **d**, **g**, **h**) staining in CX^+^/CX^−^ cells (**a**, **c**) 48 h after sham (0 Gy) and 20 Gy irradiation and 4 T1 ctrl/4 T1 Hsp70 KD cells (**b**, **d**), H1339 ctrl/H1339 HSF-1 cells (**e**, **g**), EPLC-272H ctrl/EPLC-272H HSF-1 KD cells (**f**, **h**) 48 h after sham (0 Gy) and 6 Gy irradiation, respectively. Bars represent the mean values and the corresponding standard error of the mean (SEM) of *n* = 3-4 experiments. Significance: *p ≤ 0.05, **p ≤ 0.01, ***p ≤ 0.001

For measuring radiation-induced apoptosis the percentage of active Caspase 3/7 positive cells was determined 48 h after sham (0 Gy) and 20 Gy irradiation of CX^+^/CX^−^ tumor cells by flow cytometry (Fig. 4a). After radiation exposure, the number of apoptotic cells increased in both, CX^+^ (3.11 % ± 0.06 % vs. 6.70 % ± 0.97 %, *p* ≤ 0.01) and CX^−^ (4.16 % ± 0.16 % vs. 16.67 % ± 0.81 %, *p* ≤ 0.001) cells, but, the percentage of apoptotic cells after irradiation (20 Gy) was significantly higher in CX^−^ than in CX^+^ cells (*p* ≤ 0.001). Fig. 4b shows similar results for the 4 T1 cell system 48 h after irradiation with 6 Gy. Apoptosis was significantly more pronounced in irradiated (6 Gy) compared to sham irradiated 4 T1 ctrl cells (0.60 % ± 0.06 % vs. 4.55 % ± 0.54 %, *p* ≤ 0.01) and 4 T1 Hsp70 KD cells (0.16 % ± 0.06 % vs. 7.72 % ± 0.21 %, *p* ≤ 0.001). Compared to irradiated 4 T1 ctrl cells, the percentage of apoptotic cells in 4 T1 Hsp70 KD cells with partial Hsp70 knock-down (*p* ≤ 0.001) was again significantly higher (Fig. 4b). Apoptosis as measured with the Annexin V assay in CX^+^/CX^−^ (Fig. 4c) and 4 T1 ctrl/4 T1 Hsp70 KD (Fig. 4d) tumor cells after sham (0 Gy) and 20 Gy irradiation and H1339 ctrl/H1339 HSF-1 KD and EPLC-272H ctrl/EPLC-272H HSF-1 KD cells confirmed the results of the Caspase3/7 assay. A radiation dose of 4 Gy for 4 T1 ctrl/4 T1 Hsp70 KD cells and 10 Gy for CX^+^/CX^−^ cells showed a similar trend, however, the data did not reach statistical significance (data not shown).

In contrast, lung tumor sublines H1339 ctrl/H1339 HSF-1 KD and EPLC-272H ctrl/EPLC-272H HSF-1 KD with identical mHsp70 levels did not show significant differences in apoptosis, as determined by active Caspase 3/7 and Annexin V positivity under non-irradiated (sham) and irradiated (6 Gy) conditions (Fig. 4e and f), although an increased apoptosis was observed following irradiation at 6 Gy in both tumor cell systems.

**Fig. 5:**
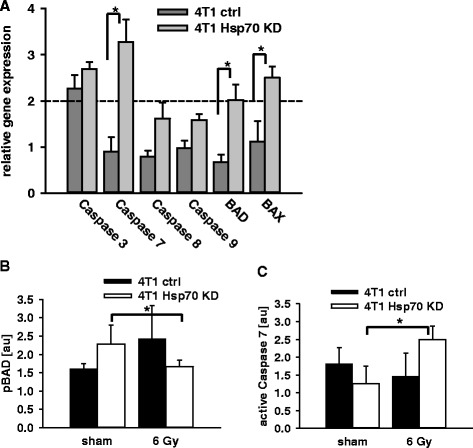
Apoptosis related gene expression of Caspase 3, Caspase 7, Caspase 8, Caspase 9, BAD and BAX were analyzed using a quantitative RT-PCR in sham (0 Gy) and 6 Gy irradiated 4 T1 ctrl/4 T1 Hsp70 KD cells (**a**) 1 h after irradiation. Bars represent the mean values and the corresponding standard error of the mean (SEM). Significance: *p ≤ 0.05. Protein expression of phosphorylated (p)BAD (**b**), active Caspase 7 (**c**) and BAX (data not shown) were determined 24 h after sham (0 Gy) and 6 Gy irradiation in 4 T1 ctrl/ 4 T1 Hsp70 KD (**b**). Significance: *p = 0.05

For further evaluation of radiation-induced changes the relative gene expression levels of the 4 T1 tumor sublines were analyzed 1 h after radiation at 0 Gy and 6 Gy. After irradiation, a more than 2-fold up-regulation in the gene expression of Caspase 3, Caspase 7, and the Bcl-2 subfamily members BAD (BH3) and BAX (Bcl-2-like protein 4) was observed in the mHsp70 low expressing 4 T1 Hsp70 KD cell line but not in the 4 T1 ctrl cell line (Fig. 5a). Significant differences in the gene expression ratios between 4 T1 ctrl and 4 T1 Hsp70 KD cells were observed for Caspase 7 (0.9-fold vs.3.3-fold, *p* ≤ 0.05), BAX (1.1-fold vs. 2.5-fold, *p* ≤ 0.05) and BAD (0.7-fold vs. 2.0-fold, *p* ≤ 0.05). No differences in the gene expression analysis were observed in 4 T1 ctrl cells that had been transfected with a control vector versus 4 T1 WT cells (data not shown).

Due to their higher radiation-resistance no significant changes in apoptosis regulated gene expression was observed in CX^+^ and CX^−^ cells after irradiation with 20 Gy (data not shown). However, also in these tumor sublines, CX^−^ cells with the lower mHsp70 expression showed an increased expression in Caspase 3/7, BAD and BAX compared to CX^+^ cells.

With respect to the levels of apoptosis related proteins differences were observed in phosphorylated (p)BAD (Fig. 5b) and active Caspase 7 (Fig. 5c), as determined by Western blot analysis. The amount of anti-apoptotic pBAD decreased in 4 T1 Hsp70 KD cells 24 h after irradiation at 6 Gy, but not in 4 T1 ctrl cells, and active Caspase 7 was found to be up-regulated following irradiation in 4 T1 Hsp70 KD cells, but not in 4 T1 ctrl cells. No significant differences were observed in the amount of the cytosolic protein BAX after irradiation (data not shown). A similar trend which did not reach statistical significance was found in the CX^+^/CX^−^ tumor sublines.

### Comparison of radiation-induced changes in the clonogenic cell survival in tumor sublines with different cytosolic and mHsp70 levels

**Fig. 6:**
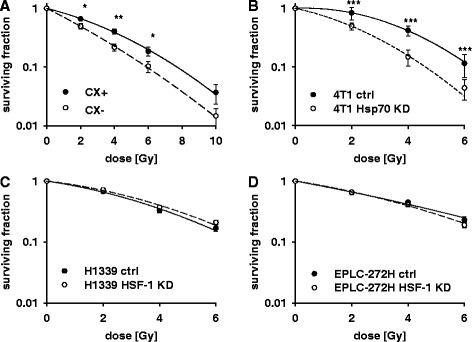
Clonogenic cell survival data of CX^+^/CX^−^ cells (**a**), (*n* = 8) following irradiation at 4 Gy, 6 Gy and 10 Gy and 4 T1 ctrl/4 T1 Hsp70 KD cells (**b**), (*n* = 3), H1339 ctrl/H1339 HSF-1 cells (**c**), (*n* = 4), EPLC-272H ctrl/EPLC-272H HSF-1 KD cells (**d**), (*n* = 4) following irradiation at 2, 4 and 6 Gy, respectively. Significance: *p ≤ 0.05, **p ≤ 0.01, ***p ≤ 0.001

Radiation sensitivity of CX (Fig. 6a), 4 T1 (Fig. 6b), H1339 (Fig. 6c) and EPLC-272H (Fig. 6d) tumor sublines was assessed in clonogenic cell survival assays. Data were fitted by the linear quadratic model (Y = (αD + βD^2^) to describe the cell survival following irradiation. In line with the data obtained by the apoptosis assays (Fig. 4a), the CX^+^ cell line, which shows a higher mHsp70 expression than the CX^−^ cell line, was significantly more radiation-resistant at 2 Gy (*p* ≤ 0.05), 4 Gy (*p* ≤ 0.01), and 6 Gy (*p* ≤ 0.05), respectively. Despite differences in the mHsp70 expression density, CX^+^ and CX^−^ tumor sublines did not differ in their cytosolic Hsp70 levels (Fig. 1). The α/β ratio was 9.67 for CX^+^ (D_50_ = 2.09 Gy) and 35.9 for CX^−^ (D_50_ = 1.45 Gy).

Figure 6b shows the results of colony forming assays for 4 T1 ctrl and 4 T1 Hsp70 KD tumor cells. In line with the data of the apoptosis assay shown in Fig. 4b, 4 T1 Hsp70 KD cells which do express very little Hsp70 in the cytosol under non-stressed conditions and exhibited a lower expression density on the membrane were significantly more radiation-sensitive than 4 T1 ctrl at each individual radiation dose (*p* ≤ 0.001, *p* ≤ 0.001 and *p* ≤ 0.001 in 2, 4, and 6 Gy, respectively). The α/β ratio was 0.6 for 4 T1 ctrl (D_50_ = 3.6 Gy) and 4.3 for 4 T1 Hsp70 KD (D_50_ = 1.9 Gy). No significant difference in radiation sensitivity was observed when clonogenic cell survival was compared in 4 T1 ctrl cells that were transfected with a control vector and 4 T1 WT cells (data not shown).

H1339 and EPLC-272H lung carcinoma cells and their mHsp70 identical HSF-1 KD counterparts did not show any significant differences in clonogenic cell survival (Fig. 6c and d) despite significant differences in their cytosolic Hsp70 levels.

## Discussion

Radiotherapy plays a central role in the therapy of solid tumors, either as a single treatment modality or in combination with surgery or systemic chemotherapy. However, therapeutic outcome is limited due to radiation-resistant tumor cell clones. Therefore, a better understanding of the biological mechanisms which are involved in radiation resistance of tumor cells might improve the outcome of clinical radiotherapy. Several biomarkers such as EpCAM [37], mutated *p53* [38] or Bcl-2 [39] have been investigated which were found to be associated to radiation sensitivity.

Hsp70 the major stress-inducible member of the HSP70 family [40] is rapidly up-regulated when cells experience stress, such as ionizing radiation, cytostatic compounds, heat and hypoxia, nutrient deficiency or any other stressful events [41–43]. It is important to note that high cytosolic levels of Hsp70 are frequently found in cancer cells even under physiological, non-stressed conditions [8, 22] and that an increased Hsp70 expression correlates with tumor progression, metastasis, resistance to cytostatic drugs and poor prognosis [8, 40, 41]. Hsp70 plays important roles in the protection against programmed cell death [10, 23] by interfering with apoptosis pathways downstream of the execution protease caspase-3-like [10], and as an inhibitor of apoptosis by the recruitment of pro-caspase 9 and binding to the apoptosome Apaf-1 [44, 45]. Together with its co-chaperone CHIP, Hsp70 can block TNFα-induced apoptosis through the formation of a complex consisting of Hsp70/CHIP and the apoptosis signal-regulating kinase 1 ASK1 [11, 46]. Furthermore, Hsp70 can inhibit the translocation of the pro-apoptotic molecule BAX to mitochondria [12]. A translocation and anchorage of Hsp70 into lysosomal membranes also has been found to promote tumor cell survival by stabilization of lysosomal membranes [22, 47]. In collaboration with the group of Jäättelä, we could show that tumor cells that do present Hsp70 on their plasma membrane also exhibit Hsp70 in their lysosomal membranes and thus are better protected towards apoptotic stimuli such as irradiation [22]. As shown previously, Hsp70 is transported to the plasma membrane via a non-classical ER/Golgi transport pathway, since inhibitors of this pathway such as Brefeldin A or Monensin did not block Hsp70 plasma membrane expression [5]. These findings are in line with earlier work of Hightower and Guidon [48] who have shown an ER-independent release of Hsp70 from viable cells with intact cell membrane. Similar to Hsp70 other molecules lacking a secretory signal such as IL1-α, IL-1β, HMGB1 are described to be exported and imported in a similar way [49]. The work of Jäättelää et al. [22] and our own data [17, 50] demonstrate a localization of Hsp70 in lysosomal membranes and endosomes. Therefore, we speculate that Hsp70 might be transported to the plasma membrane via fusion of Hsp70 carrying lipid vesicles with the plasma membrane. Potential lipid candidates which have been proven to interact with recombinant Hsp70 are phosphatidylserine PS and the lipid raft component globoyltriaosylceramide Gb3 [35, 51]. Furthermore, Hsp70 and peptides derived thereof have been found to be able to cross membranes of living tumor cells by the involvement of lipid rafts and endocytosis-dependent/independent mechanisms [16, 52, 53]. Therefore, it was assumed that after interaction of cytosolic Hsp70 with lipid vesicles containing PS or Gb3, Hsp70 can interact with these vesicles in the cytosol. After fusion of these vesicles with the plasma membrane, Hsp70 gets integrated into the plasma membrane.

Vice versa the fluorescently labelled antibody cmHsp70.1 which binds to mHsp70 on viable tumor cells translocates from the plasma membrane into early endosomes and lysosomes [17]. We hypothesize that this Hsp70-vesicle mediated translocation is reversible and also can occur from the cytosol to the membrane. Regarding these later findings herein, we asked the question as to whether plasma mHsp70 is associated with resistance of tumor cells to apoptotic cell death and thus might negatively affect sensitivity of tumor cells towards radiation [5].

Screening of more than 1000 primary human tumor biopsies and the corresponding normal tissues revealed that human carcinomas, but none of the tested corresponding normal tissues, frequently present Hsp70 on their cell surface [5–7]. Functionally, mHsp70 serves as a tumor-specific target structure for cells of the innate immune system, especially Natural Killer (NK) cells that had been activated with Hsp70 peptide TKD plus low dose IL-2. Furthermore, Hsp70 stabilizes cell membranes under stress and acts cytoprotective against apoptosis inducing mechanisms [13, 22, 54].

Although cytosolic Hsp70 has been described to exert cytoprotective properties against irradiation [27] only little is known about the role of mHsp70 in the Hsp70-mediated radiation resistance. It has been shown that mHsp70-positive tumor cells are better protected against ionizing irradiation compared to their mHsp70-negative counterparts [27]. A better understanding of the Hsp70 associated pathways which contribute to the development of radiation resistance might stimulate novel strategies to increase the radiation sensitivity of tumor cells.

To address this research question three human and one mouse isogenic tumor cell system that differed not only in their cytosolic but also in their mHsp70 levels were analyzed comparatively. Although CX^+^ tumor cells exhibited significantly higher mHsp70 expression levels than CX^−^ cells their cytosolic Hsp70 were similar [27, 28]. In contrast, the CRISPR/Cas9 Hsp70 knock-down mouse mammary carcinoma cell line 4 T1 did only express low levels of Hsp70 on the plasma membrane and in the cytosol, while 4 T1 ctrl and WT cells exhibited a strong membrane and cytosolic expression [17]. To further analyze the impact of mHsp70 in radiation resistance, Hsp70 was down-regulated in two lung carcinoma cells by a siRNA HSF-1 KD. In contrast to the CRISPR/Cas9 Hsp70 KD, an HSF-1 KD resulted in a significant decrease in cytosolic Hsp70 levels whilst mHsp70 expression remained unaffected.

Among other assays, of radiation-induced the measurement DSBs is an important endpoint for investigating radiation sensitivity because DSBs potentially lead to cell death if they are not repaired properly before start of the next cell division [55, 56]. The phosphorylation of H2A Histone family, member X (H2AX) on serine 139 which is then termed γH2AX provides a sensitive marker for DSBs. Since tumor sublines with low or missing mHsp70 expression in both isogenic cell systems (CX^−^, 4 T1 Hsp70 KD) showed higher γH2AX fluorescence intensities than their high mHsp70 expressing counterparts, irrespectively of their cytosolic Hsp70 levels we speculate that mHsp70 might be involved in the protection of tumor cells against radiation-induced stress. In contrast, tumor sublines with identical mHsp70 expression but different cytosolic Hsp70 levels did not show differences in their γH2AX values.

Radiation-induced apoptosis was analyzed using activated Caspase 3/7 antibodies and Annexin V in flow cytometry in the non-irradiated and irradiated tumor sublines. In line with the results of the үH2AX assay, significantly more apoptotic cells were found in irradiated tumor cells with a low mHsp70 expression, whereas tumor sublines with identical mHsp70 levels did not differ in apoptosis, despite differences in their cytosolic Hsp70 levels.

Apoptosis can be induced either by an intrinsic or extrinsic pathway. Both pathways depend on the initiator Caspases 2, 8, 9 and 10 and the effector Caspases 3, 6 and 7. The intrinsic pathway of apoptosis gets activated in response to various noxious stimuli, such as radiation-induced DNA damage. The balance between pro- and anti-apoptotic Bcl-2 proteins controls the permeability of the outer mitochondrial membrane [57]. BAX and BAD are both pro-apoptotic members of the Bcl-2 family and mediate the release of cytochrome *c* from the mitochondria and thus induce the intrinsic apoptotic pathway [58].

In the current study, gene expression analysis was performed with representative initiator Caspases 8, 9 and effector Caspases 3 and 7 and the pro-apoptotic molecules BAD and BAX. Following irradiation, only Hsp70 KD cells lacking Hsp70 in the cytosol and on the plasma membrane showed a significant up-regulation of effector Caspases 3, 7 and BAD and BAX. These data point towards a dependency of the intrinsic apoptotic pathway on cytosolic and mHsp70 following radiation-induced DNA damage. Although not reaching statistical significance a similar trend could be seen in the isogenic CX^+^/CX^−^ cell system. Previous findings of Gotoh and Stankiewicz [24, 25] showed that Hsp70 can block stress-induced apoptosis primarily by inhibiting the activation of BAX. In the absence of cytosolic Hsp70, BAX might be activated and thus could stimulate apoptosis. Despite differences in the BAX gene expression the cytosolic BAX protein levels did not differ significantly in the tested tumor sublines.

Traditionally, cellular radiation sensitivity is measured using the clonogenic cell survival assay as the gold standard. In line with the results of the DSB repair and apoptosis assays, the clonogenic cell survival was also significantly lower in tumor sublines with a low mHsp70 expression, irrespectively of their intracellular Hsp70 levels. These data also correspond well with the findings of Gehrmann et al. who showed that high mHsp70 expressing tumor cells are better protected against ionizing irradiation compared to their low mHsp70 expressing counterparts [27]. Moreover, patients with membrane Hsp70-positive tumors have shown a significantly decreased overall survival compared to those with mHsp70-negative tumors [59].

Our current study demonstrates that a reduction of the amount of mHsp70 might improve the possibility to induce apoptosis through the intrinsic apoptotic pathway after X-ray irradiation. This finding is in line with previous reports showing that chemotherapy and radiotherapy predominantly initiate apoptosis through the intrinsic pathway [20]. By using an HSF-1 KD system which down-regulates cytosolic but not mHsp70 levels we could demonstrate that mHsp70 but not cytosolic Hsp70 levels have an impact on the radiation sensitivity of tumor cells. This is an important finding for the development of novel radiotherapy strategies for the treatment of radiation resistant tumor cells. Future studies assessing radiation-sensitization with inhibitors of the vesicular transport of Hsp70 to the plasma membrane of tumor cells could be taken into account. Furthermore, the mHsp70 status which could be determined in the serum of patients using a novel ELISA detecting liposomal Hsp70 which is actively released by mHsp70 positive viable tumor cells [60] might provide a useful tool to predict the radiation sensitivity of tumors over time. We have shown previously that the majority of Hsp70 found in the circulation of tumor patients is actively released in lipid vesicles. Patients with inflammatory diseases have significantly lower serum Hsp70 levels than tumor patients [61]. In these patients Hsp70 is released as a free protein which is predominantly derived from dying cells. Therefore, we speculate that significantly elevated Hsp70 serum levels which are detectable by the novel lipHsp70 ELISA [59] might have diagnostic/prognostic value.

## Conclusion

The results of this study indicate that not only cytosolic but also mHsp70 has an impact on radiation sensitivity. Thus, mHsp70 expression is associated with defined variations in the regulation of survival pathways in response to radiation. The expression of mHsp70 on tumor cells should be considered as potential new biomarker to identify radiation-resistance of tumor cells.

## References

[1] Botzler C, Ellwart J, Günther W, Eissner G, Multhoff G (1999). Synergistic effects of heat and ET-18-OCH3 on membrane expression of hsp70 and lysis of leukemic K562 cells. Exp Hematol.

[2] Sharma S, Singh R, Kaur M, Kaur G (2010). Late-onset dietary restriction compensates for age-related increase in oxidative stress and alterations of HSP70 and synapsin 1 protein levels in male Wistar rats. Biogerontology.

[3] Ciocca DR, Arrigo AP, Calderwood SK (2013). Heat shock proteins and heat shock factor-1 in carcinogenesis and tumor development: an update. Arch Toxicol.

[4] Dakappagari N, Neely L, Tangri S, Lundgren K, Hipolito L, Estrellado A (2010). An investigation into the potential use of serum Hsp70 as a novel tumour biomarker for Hsp90 inhibitors. Biomarkers.

[5] Multhoff G, Botzler C, Wiesnet M, Müller E, Meier T, Wilmanns W (1995). A stress-inducible 72-kDa heat-shock protein (HSP72) is expressed on the surface of human tumor cells, but not on normal cells. Int J Cancer.

[6] Ferrarini M, Heltai S, Zocchi MR, Rugarli C (1992). Unusual expression and localization of heat-shock proteins in human tumor cells. Int J Cancer.

[7] Hantschel M, Pfister K, Jordan A, Scholz R, Andreesen R, Schmitz G (2000). Hsp70 plasma membrane expression on primary tumor biopsy material and bone marrow of leukemic patients. Cell Stress Chaperones.

[8] Aghdassi A, Phillips P, Dudeja V, Dhaulakhandi D, Sharif R, Dawra R (2007). Heat shock protein 70 increases tumorigenicity and inhibits apoptosis in pancreatic adenocarcinoma. Cancer Res.

[9] Garrido C, Gurbuxani S, Ravagnan L, Kroemer G (2001). Heat shock proteins: endogenous modulators of apoptotic cell death. Biochem Biophys Res Commun.

[10] Jäättelä M, Wissing D, Kokholm K, Kallunki T, Egeblad M (1998). Hsp70 exerts its anti-apoptotic function downstream of caspase-3-like proteases. EMBO J.

[11] Gao Y, Han C, Huang H, Xin Y, Xu Y, Luo L (2010). Heat shock protein 70 together with its co-chaperone CHIP inhibits TNF-alpha induced apoptosis by promoting proteasomal degradation of apoptosis signal-regulating kinase1. Apoptosis.

[12] Yang X, Wang J, Zhou Y, Wang Y, Wang S, Zhang W (2012). Hsp70 promotes chemoresistance by blocking Bax mitochondrial translocation in ovarian cancer cells. Cancer Lett.

[13] Kirkegaard T, Roth AG, Petersen NH, Mahalka AK, Olsen OD, Moilanen I (2010). Hsp70 stabilizes lysosomes and reverts Niemann-Pick disease-associated lysosomal pathology. Nature.

[14] Bayer C, Liebhardt ME, Schmid TE, Trajkovic-Arsic M, Hube K, Specht HM (2014). Validation of heat shock protein 70 as a tumor-specific biomarker for monitoring the outcome of radiation therapy in tumor mouse models. Int J Radiat Oncol Biol Phys.

[15] Gehrmann M, Specht HM, Bayer C, Brandstetter M, Chizzali B, Duma M (2014). Hsp70 - a biomarker for tumor detection and monitoring of outcome of radiation therapy in patients with squamous cell carcinoma of the head and neck. Radiat Oncol.

[16] Stangl S, Varga J, Freysoldt B, Trajkovic-Arsic M, Siveke JT, Greten FR (2014). Selective in vivo imaging of syngeneic, spontaneous, and xenograft tumors using a novel tumor cell-specific Hsp70 peptide-based probe. Cancer Res.

[17] Gehrmann M, Stangl S, Foulds GA, Oellinger R, Breuninger S, Rad R (2014). Tumor imaging and targeting potential of an Hsp70-derived 14-mer peptide. PLoS One.

[18] Rerole AL, Jego G, Garrido C (2011). Hsp70: anti-apoptotic and tumorigenic protein. Methods Mol Biol (Clifton, NJ).

[19] Sayers TJ (2011). Targeting the extrinsic apoptosis signaling pathway for cancer therapy. Cancer Immunol Immunother.

[20] Igney FH, Krammer PH (2002). Death and anti-death: tumour resistance to apoptosis. Nat Rev Cancer.

[21] Kulikov AV, Shilov ES, Mufazalov IA, Gogvadze V, Nedospasov SA, Zhivotovsky B (2012). Cytochrome c: the Achilles’ heel in apoptosis. Cell Mol Life Sci.

[22] Nylandsted J, Gyrd-Hansen M, Danielewicz A, Fehrenbacher N, Lademann U, Hoyer-Hansen M (2004). Heat shock protein 70 promotes cell survival by inhibiting lysosomal membrane permeabilization. J Exp Med.

[23] Jäättelä M (1999). Escaping cell death: survival proteins in cancer. Exp Cell Res.

[24] Gotoh T, Terada K, Oyadomari S, Mori M (2004). hsp70-DnaJ chaperone pair prevents nitric oxide- and CHOP-induced apoptosis by inhibiting translocation of Bax to mitochondria. Cell Death Differ.

[25] Stankiewicz AR, Lachapelle G, Foo CP, Radicioni SM, Mosser DD (2005). Hsp70 inhibits heat-induced apoptosis upstream of mitochondria by preventing Bax translocation. J Biol Chem.

[26] Guo F, Sigua C, Bali P, George P, Fiskus W, Scuto A (2005). Mechanistic role of heat shock protein 70 in Bcr-Abl-mediated resistance to apoptosis in human acute leukemia cells. Blood.

[27] Gehrmann M, Marienhagen J, Eichholtz-Wirth H, Fritz E, Ellwart J, Jäättelä M (2005). Dual function of membrane-bound heat shock protein 70 (Hsp70), Bag-4, and Hsp40: protection against radiation-induced effects and target structure for natural killer cells. Cell Death Differ.

[28] Multhoff G, Botzler C, Jennen L, Schmidt J, Ellwart J, Issels R (1997). Heat shock protein 72 on tumor cells: a recognition structure for natural killer cells. J Immunol.

[29] Zaarur N, Gabai V, Porco J, Calderwood S, Sherman M (2006). Targeting heat shock response to sensitize cancer cells to proteasome and Hsp90 inhibitors. Cancer Res.

[30] Pulaski BA, Terman DS, Khan S, Muller E, Ostrand-Rosenberg S (2000). Cooperativity of Staphylococcal aureus enterotoxin B superantigen, major histocompatibility complex class II, and CD80 for immunotherapy of advanced spontaneous metastases in a clinically relevant postoperative mouse breast cancer model. Cancer Res.

[31] Jinek M, Chylinski K, Fonfara I, Hauer M, Doudna JA, Charpentier E (2012). A programmable dual-RNA-guided DNA endonuclease in adaptive bacterial immunity. Science.

[32] Pouget JP, Mather SJ (2001). General aspects of the cellular response to low- and high-LET radiation. Eur J Nucl Med.

[33] Ran FA, Hsu PD, Lin CY, Gootenberg JS, Konermann S, Trevino AE (2013). Double nicking by RNA-guided CRISPR Cas9 for enhanced genome editing specificity. Cell.

[34] Laemmli UK (1970). Cleavage of structural proteins during the assembly of the head of bacteriophage T4. Nature.

[35] Schilling D, Gehrmann M, Steinem C, De MA, Pockley AG, Abend M (2009). Binding of heat shock protein 70 to extracellular phosphatidylserine promotes killing of normoxic and hypoxic tumor cells. FASEB J.

[36] Towbin H, Staehelin T, Gordon J (1979). Electrophoretic transfer of proteins from polyacrylamide gels to nitrocellulose sheets: procedure and some applications. Proc Natl Acad Sci U S A.

[37] Murakami N, Mori T, Yoshimoto S, Ito Y, Kobayashi K, Ken H (2014). Expression of EpCAM and prognosis in early-stage glottic cancer treated by radiotherapy. Laryngoscope.

[38] Lee JM, Bernstein A (1993). p53 mutations increase resistance to ionizing radiation. Proc Natl Acad Sci U S A.

[39] Wu H, Schiff DS, Lin Y, Neboori HJ, Goyal S, Feng Z (2014). Ionizing radiation sensitizes breast cancer cells to Bcl-2 inhibitor, ABT-737, through regulating Mcl-1. Radiat Res.

[40] Zhang P, Leu JI, Murphy ME, George DL, Marmorstein R (2014). Crystal structure of the stress-inducible human heat shock protein 70 substrate-binding domain in complex with peptide substrate. PLoS ONE.

[41] Daugaard M, Rohde M, Jäättelä M (2007). The heat shock protein 70 family: Highly homologous proteins with overlapping and distinct functions. FEBS Lett.

[42] Daugaard M, Jäättelä M, Rohde M (2005). Hsp70-2 is required for tumor cell growth and survival. Cell Cycle.

[43] Zorzi E, Bonvini P (2011). Inducible hsp70 in the regulation of cancer cell survival: analysis of chaperone induction, expression and activity. Cancer.

[44] Beere HM, Wolf BB, Cain K, Mosser DD, Mahboubi A, Kuwana T (2000). Heat-shock protein 70 inhibits apoptosis by preventing recruitment of procaspase-9 to the Apaf-1 apoptosome. Nat Cell Biol.

[45] Pandey P, Saleh A, Nakazawa A, Kumar S, Srinivasula SM, Kumar V (2000). Negative regulation of cytochrome c-mediated oligomerization of Apaf-1 and activation of procaspase-9 by heat shock protein 90. EMBO J.

[46] Park HS, Cho SG, Kim CK, Hwang HS, Noh KT, Kim MS (2002). Heat shock protein hsp72 is a negative regulator of apoptosis signal-regulating kinase 1. Mol Cell Biol.

[47] Petersen NH, Kirkegaard T, Olsen OD, Jäättelä M (2010). Connecting Hsp70, sphingolipid metabolism and lysosomal stability. Cell Cycle.

[48] Hightower LE, Guidon PT (1989). Selective release from cultured mammalian cells of heat-shock (stress) proteins that resemble glia-axon transfer proetisn. J Cell Physiol.

[49] Nickel W, Seedorf M (2008). Unconventional mechansims of proetein transport to the cell surface of eukaryotic cells. Annu Rev Cell Dev Biol.

[50] Multhoff G (2007). Heat shock protein 70 (Hsp70): membrane location, export and immunological relevance. Methods.

[51] Gehrmann M, Liebisch G, Schmitz G, Anderson R, Steinem C, De MA (2008). Tumor-specific Hsp70 plasma membrane localization is enabled by the glycosphingolipid Gb3. PLoS ONE.

[52] Komarova EY, Meshalkina DA, Aksenov ND, Pchelin IM, Martynova E, Margulis BA (2015). The discovery of Hsp70 domain with cell-penetrating activity. Cell Stress Chaperones.

[53] Shevtsov MA, Komarova EY, Meshalkina DA, Bychkova NV, Aksenov ND, Abkin SV (2014). Exogenously delivered Hsp70 displaces its endogenous analogue and sensitizes cenacer cells to lymphyocytes-mediated cytotoxicity. Oncotarget.

[54] Horvath I, Vigh L (2010). Cell biology: Stability in times of stress. Nature.

[55] Olive PL, Banath JP, Sinnott LT (2004). Phosphorylated histone H2AX in spheroids, tumors, and tissues of mice exposed to etoposide and 3-amino-1,2,4-benzotriazine-1,3-dioxide. Cancer Res.

[56] Olive PL, Banath JP (2004). Phosphorylation of histone H2AX as a measure of radiosensitivity. Int J Radiat Oncol Biol Phys.

[57] Tait SW, Green DR (2010). Mitochondria and cell death: outer membrane permeabilization and beyond. Nat Rev Mol Cell Biol.

[58] Korsmeyer SJ, Wei MC, Saito M, Weiler S, Oh KJ, Schlesinger PH (2000). Pro-apoptotic cascade activates BID, which oligomerizes BAK or BAX into pores that result in the release of cytochrome c. Cell Death Differ.

[59] Pfister K, Radons J, Busch R, Tidball JG, Pfeifer M, Freitag L (2007). Patient survival by Hsp70 membrane phenotype: association with different routes of metastasis. Cancer.

[60] Breuninger S, Ertl J, Bayer C, Knape C, Gunther S, Regel I, et al. Quantative analysis of liposomal Hsp70 in the blood of tumor patients using a novel lipHsp70 ELISA. J Clin Cell Immunol. doi:org/10.4172/2155-9899.1000264.

[61] Gehrmann M, Cervello M, Montalto G, Capello F, Gulino A, Knape C (2014). Hsp70 serum levels differ significantly in patients with chronic hepatitis, lievr chirrosis and hepatocellular carcinoma. Front Immunol.

